# Corporate social responsibility & students’ perceptions: evidence from Indian higher education institutions

**DOI:** 10.12688/f1000research.137572.1

**Published:** 2023-09-22

**Authors:** Premendra Kumar Singh, Raju Ganesh Sunder, Pravin Narayan Mahamuni, Elangbam Nixon Singh, Bidhu Kanti Das, Anand G. Jumle, Rajeshkumar Kashayp

**Affiliations:** 1 School of Online Education, Bharati Vidyapeeth (Deemed to be University), Pune, Maharashtra, 411030, India; 2 Center for Distance and Online Education, Datta Meghe Institute of Higher Education and Research, Wardha, Maharashtra, 442001, India; 3 Symbiosis School for Online and Digital Learning, Symbiosis International University, Pune, Maharashtra, 412115, India; 4 Finance Officer, Manipur University, Imphal, Manipur, 795003, India; 5 Department of Management, Mizoram University, Aizawl, Mizoram, 796004, India; 6 Indira College of Art, Commerce & Science, Pune, Maharashtra, 411033, India; 7 Sadhu Vaswani Institute of Management Studies for Girls, Pune, Maharashtra, 411041, India

**Keywords:** Corporate Social Responsibility, Perceptions, Higher Educational Institutes.

## Abstract

**Background:** For many, the understanding of Corporate Social Responsibility (CSR) may sound a rather new topic, but it has been pondered upon by great thinkers of the world for many decades. The initial form of CSR was more of philanthropic which has become more of mandatory norm in Indian context. We believe the future of a nation are the youth of the nation and their perceptions on the matter of CSR are of great importance. Therefore, the purpose of the current study is to investigate the many viewpoints that students have about CSR and to determine whether or not socioeconomic characteristics (gender, age, professional experience, and academic degree) influence these views.

**Methods:** The research methodology comprises of utilization of an accepted scale ( PRESOR) for collecting data of perception on CSR. Data was collected using an online questionnaire, distributed to students at Higher Educational Institutes of Northeast India. Responses from 272 students were received out of which we rejected unengaged responses of 25 students, and we continued with responses from 247 students. We have utilized Factor Analysis, Multivariate analysis of variance (MANOVA), & t-test for the scrutinizing the collected data.

**Results:** The perceptions of students show a variety of dimensions, which may be categorised as: (a) value CSR, (b) against CSR, and (c) neutral to CSR. It is also found that the sociodemographic variables have a statistically significant influence on students' notion of CSR.

**Conclusions:** This study is one of the first investigative works that has utilized modified PRESOR model for examining the perception of CSR in Indian context. The model was found to be fit to be used in Indian context. The study concluded that sociodemographic variables such as Age, Education, Professional Experience influences perception of CSR.

## Introduction

Corporate social responsibility (CSR) has received symbolic recognition in the eyes of corporations, customers, and academic professionals due to the considerable societal consequences that corporations have ( Džupina, 2016; Ortiz-Avram *et al.,* 2018; Schmidt and Cracau, 2018). Companies evolving progressively are mindful of the role of corporate social responsibility (CSR) in terms of global competitiveness ( Petrović-Ranđelović *et al*., 2015). This understanding allows them to concentrate not just on delivering profits, but also to make informed choices that are morally & socially acceptable to all concerned stakeholders, such as the community, the climate, and investors ( Bir *et al.,* 2009; Teixeira *et al.,* 2018).

Regardless of the fact that there has been extensive research on CSR for more than five decades, ( *e.g.*, Bowen, 1953; Davis, 1973), many academicians maintain to be interested in this area ( *e.g.*, Burton & Goldsby, 2009; Ortiz-Avram *et al.,* 2018). Despite this, many respected academics have moved their attention from CSR to the achievement of corporations in the sphere of social responsibility ( Wartick and Cochran, 1985) and how it affects the company's overall success ( Ciampi, 2018; Waddock and Graves, 1997) and if CSR plays role in customer loyalty ( Park and Kim, 2019). According to Maignan (2001), concerns pertaining to CSR, on the other hand, continue to be a subject of research that receives little attention. This is especially true with research on the perception that students have about CSR, particularly in the Indian context.

In simple terms, social responsibility may be viewed as a collection of a course of action adopted by corporations to boost economic, societal, and environmental situations, efforts that go above and beyond the requirements of law ( Godfrey *et al.,* 2009). As a result, given the fact that responsibilities towards the society are gaining more and more significance in the corporate world, it is essential to explore the opinions of workers, executives, and entrepreneurs, or, more specifically, the perceptions of Higher Education Institutions (HEIs) students, on this aspect ( Teixeira *et al.,* 2018).

A person’s positive, negative or neutral perceptions of CSR are influenced by culture and the nation they are brought up in ( Usunier and Lee, 2013). Available literature also shows that students from various cultural backgrounds have varied perspectives on CSR ( Akbaş *et al.,* 2012; Pätäri *et al.,* 2017; Wong *et al.,* 2010). The intention of this paper is to contemplate if students of India have different perceptions on understanding of CSR and also to substantiate if sociodemographic factors instigate students’ perception on the area of CSR.

This paper initiates itself with a literature review indicating the applicability of the topic in Higher Education Institution (HE). This also comprises of the various important definition of CSR, the importance and actions leading to socially responsible actions apart from set of hypotheses and purpose of studying the students’ perception on CSR. These are addressed by utilizing questions used earlier in a study which assessed students’ notion of corporate social responsibility ( Fitzpatrick, 2013; Teixeira *et al.,* 2018).

This study is conducted to investigate the perception by taking a representative sample of students from Indian Higher Education Institutions by employing the model proposed by Fitzpatrick (2013) and Teixeira *et al.* (2018) and utilized various univariate and multivariate statistical techniques that were most appropriate for the study's objectives.

CSR becomes a crucial business choice as a result of globalization's influence on the business, climatic and economic development ( McGuire *et al.,* 1988) so that companies can meet the expectations of a wider range of customers and stakeholders ( Dahlsrud, 2008). Corporations that practise corporate social responsibility (CSR) recognise that their actions have consequences for a wide range of people and places, including consumers, suppliers, workers, shareholders, communities, and the climate ( Wymer and Rundle-Thiele, 2017). Although CSR is a hot issue, there are no mutual agreement on how the concept should be defined. This is because various scholars propose different definitions based on different aspects, with just a few sharing anything in common ( Dahlsrud, 2008).

CSR seems like it ought to be part of the "triple bottom line," which holds that an organization’s success is determined by three factors: its financial health, its commitment to the environment, and its impact on society ( Zadek *et al.,* 2003). Thus, CSR has the potential to initiate corporate social progress, which can come in a variety of forms. Some of these forms include internal changes regarding manufacturing methods, minimised environmental impacts, increases in satisfaction among employees, and new interactions along the chain. Investments in the building infrastructure of local communities and growth of community-based activities are also possible outcomes of CSR ( Aguilera *et al.,* 2007).

Therefore, companies need to integrate social consciousness into their overarching strategies by doing things like differentiating their goods depending on the social traits they possess. This will increase brand loyalty and establish the business as a trustworthy and ethical option for consumers ( Siegel and Vitaliano, 2007).

The number of socially responsible investment funds has grown, and so has the amount of money invested in them, as noted by Fitzpatrick (2013, p. 86). CSR is a widely debated issue in the commercial and academic spheres because of its pervasiveness in today's global economy ( Doh and Guay, 2006). Carroll (2016) argues that creativity, market distinctiveness, enthusiastic staff participation are the reasons for companies to adopt CSR whereas Kurucz *et al.*, 2009, suggest that cost and risk reductions, enhancement of competitive advantage, bolstering of firm legitimacy and reputation, and the creation of win-win scenarios for both the company and society are the reason why companies must accept and work towards the advancement of CSR. Companies should focus on producing value rather than merely following current procedures since there is a risk that huge corporations would engage in unethical behaviour in their pursuit of profit maximisation ( Santos, 2012).

Although CSR has not yet permeated every sector of industry ( Sánchez-Hernández and Mainardes, 2016), this topic is focus of fresh research from a wide range of institutions, most notably, Higher Education Institutes (HEI) ( Vallaeys *et al.,* 2009; Vázquez *et al.,* 2014, 2016). Vallaeys *et al*., (2009), defined CSR as competent leadership in all areas of educational institutions at a higher level (teaching, research, extension, and administration) is central to this approach. As a result, several universities' business programmes now emphasise ethical behaviour and accountability to society topics in their curriculum to better meet the needs of businesses and the community ( Assudani *et al.,* 2011).

In regard to Higher Education Institutions, CSR must include all phases of an institution's teaching, research, and knowledge transfer activities, including planning, design, implementation, and evaluation ( Esfijani *et al.,* 2013). The terminology "University Social Responsibility" (USR) refers to collective responsibilities of Higher Education Institutions in areas such as Increasing access to higher education, creating market-relevant curriculum, producing high-quality graduates, reducing waste, bolstering research capacity, and meeting other societal demands are all goals of changing the academic system ( Esfijani *et al.,* 2013). Institutions committed to morality, good administration, and ecological consciousness, social participation, and the promotion of values; HEIs shape society through educating its members and expanding their horizons via the dissemination of information ( Vallaeys *et al.,* 2009).

Therefore, higher education institutions (HEI) are crucial in producing ethical and socially conscious business leaders ( Alsop, 2006), and because of this reason an increase courses devoted to CSR is observed ( Wymer and Rundle-Thiele, 2017). Sánchez-Hernández & Mainardes (2016) note that a combination of factors—including increased competitiveness and shifting demographics—has resulted in higher education institutions (HEIs) being increasingly student-centric.

### Students’ perceptions of corporate social responsibility

Given that corporate social responsibility (CSR) has grown in importance and relevance in the corporate world, studying the views of today's students—the employees, employers, and company owners of tomorrow - on the topic is intriguing ( Assudani *et al.,* 2011, p. 103). As a result, HEIs should aid in the preparation of socially responsible persons by emphasising the significance of upholding ethical standards in the workplace ( Wymer and Rundle-Thiele, 2017). It is because, according to these authors, “education on ethical topics increases moral cognition and deliberation” ( Lau, 2010).

There has been some empirical work done to quantify students' a conscience of duty to others ( Elias, 2004), several categories and dimensions can be identified as a result ( González-Rodríguez *et al.,* 2013). Despite widespread agreement that CSR helps businesses thrive and raises their profile in the public eye, and despite CSR's maturation into a useful strategic tool, opinions on it among students are split ( Mcguire *et al.,* 1988; Schmidt & Cracau, 2018; Ugwuozor, 2020)).

The distinct aspects of the social responsibility identified included charity ( Akbaş *et al.,* 2012; Wong *et al.,* 2010); statutory mandates or duty to obey the law ( Akbaş *et al.,* 2012; Arli *et al.,* 2014a; Wong *et al.,* 2010); edge over competitors through innovation and continuous learning; economic & ethical commitment ( Akbaş *et al.,* 2012; Arli *et al.,* 2014a). Also, studies employing the PRESOR instrument reveal that profit, longevity, and rapid growth are all characteristics of ethical behaviour ( Elias, 2004; Fitzpatrick, 2013; Singhapakdi *et al.,* 1996).

However, when more instances of corporate hypocrisy are brought to light, customers acquire a natural mistrust towards the CSR promises made by businesses ( Connors *et al.,* 2017). This is even though consumers' perspectives could vary since CSR has developed at various rates in Western countries and in Eastern countries, and even within regions ( González-Rodríguez *et al.,* 2013). As a result, certain aspects of CSR views that are identified in the literature that are not in a good light. One such aspect is the prevalent assumption that businesses may enhance their image if they are sensitive to concerns raised by the public ( Wals, 2007). Therefore, CSR may be perceived as a manufactured performance; in addition, students who embrace the principles of altruism are more likely to have good opinions of CSR than students who adopt self-centred values ( Pätäri *et al.,* 2017).

Therefore, the plethora of previously listed notions proves that there is no mutual acceptance on how CSR ought to be defined as the preceding examples of overlapping ideas, investigation of the likelihood that there is a plurality of elements in this material might benefit by identifying the many characteristics that influence students' views of CSR. Within this context, we propose the following hypothesis in light of the research demonstrating that students' attitudes of corporate social responsibility are influenced by their nationality ( Pätäri *et al.,* 2017; Wong *et al.,* 2010) as well as the fact that the study conducted by Fitzpatrick (2013) at an American higher education institution did not investigate the many facets that influence students' views of CSR.

Hypothesis 1: Students’ perceptions of CSR can be considered in different dimensions, including having supporting perception about CSR, negative perception or maybe neutral positioning.

In conjunction with the uniqueness of each nation's culture and set of natural advantages, sociodemographics are taken into account as a factor that might affect how people view CSR and ultimately shape their moral judgements ( Arli *et al.,* 2014b; Fitzpatrick, 2013; González-Rodríguez *et al.,* 2013; Wong *et al.,* 2010). When examining students' perspectives on CSR, we may generalise about the effects of sociodemographic factors play a role in shaping how students feel about the organization's commitment to social and environmental responsibility ( Fitzpatrick, 2013; Ugwuozor, 2020). These studies allow us to formulate the suggested hypothesis:

Hypothesis 2: Sociodemographic students’ characteristics can influence their perception of CSR

## Methods

As the research was conducted on the students in order to measure their perceptions, necessary approval was taken from the ethical committee. The Human Ethics Committee of Mizoram University, India in their letter, bearing the reference number MZU/HEC/2023/011, dated 04.07.2023 reviewed the questionnaire and proposal and approved the collected data for further processing.

The questionnaire, which was provided to the students, clearly mentioned that the “The information given by student will be used for academic purpose only” and the students agreeing to this statement have filled up the questionnaire.

In order to determine whether or not the hypotheses proposed are valid, a quantitative approach was utilised. The motive is to have an insight and determine whether students’ perceptions in the present study may be linked to other variables and to assess the influence of sociodemographic attributes in their perceptions of issues pertaining to CSR and other social concerns.

For measuring the students’ perceptions, a slightly modified version of PRESOR, which was initially designed to assess the views of marketers’ perception with relation to the significance in terms of morality and civic duty ( Singhapakdi *et al.,* 1996) and was then used by ( Fitzpatrick, 2013; Teixeira *et al.,* 2018) to measure the perception of students about CSR was adopted.

Perceived Role of Ethics and Social Responsibility (PRESOR), a measurement having three major dimensions namely Social Responsibility and Profitability, Long Term and Short-Term Gains was developed with potentials of supplementing in making concrete decision in situations which involves ethical and social issues ( Singhapakdi *et al.,* 1996). The scale was modified and was adapted to investigate how gender, job experience, and spirituality impact students' opinions of corporate social responsibility (CSR) ( Fitzpatrick, 2013). On the basis of the same adapted scale ( Teixeira *et al.,* 2018), investigated the many views that students hold on CSR, therefore giving a platform with an accepted scale for measurement of CSR perceptions which are shown in **Table 2, underlying data** and on basis of prior research, the relations that are mentioned in Table 1, **underlying data** was expected to be found. All Tables can be found as *Underlying data* ( Sunder, 2023).

The data was collected from students of Master of Business Administration (MBA) & Bachelor of Business Administration (BBA) from the various reputed Central Universities, State Universities and Private Universities in the North-eastern States of India between April to September 2022. Online Survey method was deployed as it can have a larger reach and higher number of responses could be collected. Responses from 272 students were received out of which 25 responses were filtered out because of unengaged responses and we continued with a total of 247 responses which were collected by convenience sampling through a well-structured 16 questions Likert scale-based survey plus five demographic questions.

The data that was obtained was evaluated employing IBM SPSS statistics version 21, included both univariate analyses, such as descriptive analysis, t-tests, and Pearson tests, and multivariate analyses, which included confirmatory factor analysis and Multivariate analysis of variance (MANOVA), were performed. The selection of the aforementioned statistical methods was guided by the proposed study framework as depicted in **Table 1, underlying data** ( Sunder, 2023).

**Table 3, underlying data** ( Sunder, 2023) represent while most students (89.1%) were in the age range 17 and 26, responses came from students as young as 17 and as old as 50. It was estimated that about 93% of the students were unmarried, while the residual 7% were married. Most respondents (54.3%) had some bachelor's degree, while 4.9% having earned a master's degree and 40.9% obtained professional courses. Regarding employment, 75.3% of respondents were enrolled as full-time students while 12.1% were working full-time (student worker).

## Results

Complementary to study of Fitzpatrick, (2013) and Teixeira *et al.,* (2018), Table 2, underlying data takes into account and compiles students' judgment of CSR in this study ( Sunder, 2023).

The same table presents the means and is viable to deduce that statements with the higher means are the statements which support CSR, for instance, perceptions 15, 6, 4, 1 and 11. On the contrary, perceptions 5, 8 and 13, with lower average suggests that CSR is not a significant choice that companies should address.

Taking into consideration the above findings, Factor Analysis was conducted on the perception of the students about CSR to deduce the dimensions to meet our first objective of the study.

The value of Kaiser-Meyer-Olkin (KMO) computed was 0.914, which indicates that the sample size is suitable for carrying out factor analysis. On top of that, significance level of the Bartlett Test is 0 (0.000), which means that the sample is suitable for this method at a significance level of 1%. Table 4, underlying data shows the results, and in particular when individual MSA values are considered, it becomes clear that the samples were sufficiently representative, as there is no parameter with an MSA score below 0.5 ( Sunder, 2023). As a consequence, all of these factors are deemed crucial to the investigation, and they have enough properties with the order variables for viability metrics to be produced from them. The values of the rotated components demonstrate that the variables are associated to one another, with the three extracted constructs having a variation explained by the factors that accounts for about 56% of the variance of the overall variance involving the variables. Using the factor analysis shown in Table 4, underlying data ( Sunder, 2023) and drawing on earlier explanation of PRESOR ( Fitzpatrick, 2013; Singhapakdi *et al.,* 1996), we may deduce that there are three distinct dimensions at play here: component 1 contains positive views of CSR (averaging 5.16), component 2 comprised negative perceptions of CSR (averaging 4.16), and factor 3 comprises neutral perceptions of CSR (averaging 4.68).

Since the values in **Table 4** are more than 0.60 ( Sunder, 2023), suggesting that the measures are trustworthy, a reliability study utilising the Cronbach's Alpha coefficient may be employed to validate the results of the confirmatory factorial analysis ( Nunnally and Bernstein, 1994; Pallant, 2001). As the questions were adopted, we did not do exploratory factor analysis, but to see if the model fits a different geographical location, Confirmatory Factor Analysis was conducted by making use of IBM SPSS Amos version 21 software to evaluate the model fit by utilizing the plugin "Model Fit Measure" ( Gaskin and Lim, 2016). A satisfactory match with the model was observed by various indicatives namely CFI = 0.936; CMIN/DF = 2.512; RMSEA = 0.078; SRMR = 0.066 which is in line with the cutoff criteria of good fit values suggested by Hu & Bentler, (1999).

Because of this, it is feasible to draw the conclusion, on the basis of the findings of the factor analysis, that the results of these views demonstrate that there are several ways in which students understand CSR, and that CSR is not universally seen as a major regard for businesses. Therefore, the first goal was successfully completed, and hypothesis 1 was verified.

To understand if the sociodemographic variables taken in the research have any impact on the notion of students about CSR, Pearson Corelation investigation into whether or not any corelation reside between the variables under study. Students' views on CSR were broken down into their constituent dimensions through factor analysis, and a correlation test was run between these elements and sociodemographic factors. The corelation test at 5% significance level reflects relationship between the dimensions of student perception about CSR and the sociodemographic characteristics which is contrary to the findings of ( Teixeira *et al.,* 2018). Positive corelation is observed between the three dimensions of CSR: positive attitudes towards CSR, attitudes that cast doubt on the significance of CSR, and the prioritisation of business activities that push CSR to the back burner and the sociodemographic characteristics in the context of Indian students. It is not statistically significant that if there is any corelation between variable age and civil Status and the three dimensions of CSR perception.

In order to address to the other hypotheses which were related to the sociodemographic variables, we used MANOVA for those hypotheses testing where the independent variables were Age, Academic Degree and Experience and t-test was conducted on the variables which had only two options namely Gender and Civil Status. The use of MANOVA was preferred over using multiple ANOVAs to test statistical significance difference between several groups ( Maroco, 2003). For the purpose of MANOVA, the reduced dimensions were used as the dependent variables and the sociodemographic variables were considered as independent variables. But before the hypotheses were tested, assumptions of multivariate analysis of variance (MANVOA) namely multivariate normality, homoscedasticity and existence of corelation between the independent variables were fulfilled as depicted in **Table 5** ( Sunder, 2023).

Addressing hypothesis 2.1, "The influence of age on students' perceptions of CSR," the Wilks' Lambda = 0.907, F (15, 660) = 1.578, p = 0.074, illustrates that the dependent variable has a statistically significant impact on the independent factors at a significance level of 10%. However, the Test of Between Subjects Effects reflects that the first reduced dimension is significant at 10% level, instead of one-way ANOVA we did a Levene’s test of equality of error variances and it was found to be insignificant. We did a Scheffe Post-hoc test to confirm the findings and it was confirmed that there was no significant difference between age interval and the reduced dimensions. As all the values are greater than 0.10, the results show no significant differences across age groups. Therefore, we conclude that age positively influences students’ perception of CSR.

For Hypothesis 2.2, we used MANOVA again and the values of Wilks' Lambda = 0.951, F (6, 484) = 2.032, p = 0.060, indicates that the dependent variable has a statistically significant impact on the independent factors at a significance level of 10%. Levene’s test was conducted and it was found to be insignificant, which meets the presumption of homogeneity of variance. The output of the Test of Between Subjects Effects reflects significant difference between second and third reduced dimension. Based on the contrast results upon comparison of undergraduate and professional technical courses there was significant difference in all the three reduced dimensions. Therefore hypothesis 2.2 *i.e.,* “academic degree positively influences the students’ perceptions of CSR” is accepted.

For the hypothesis 2.3, we continued with MANOVA and it was observed that, the Wilks' Lambda = 0.919, F (12, 635) = 1.515, p = 0.060 was observed which implies that the dependent variables have statistically significant effect on the independent variables at 10% significance level. Besides that, the Levene’s test (used in place of ANOVA) reflets insignificant values and the p-values of Test of Between Subjects Effects were all insignificant implying that the hypothesis is accepted: “professional experience positively influence the students’ perception of CSR”.

To test hypothesis 2.4 and 2.5, We conducted t-tests to look at whether or not there is a correlation amongst students' views on CSR and their gender, civil status, comparison is made between the means of the two groups taking into consideration three reduced dimensions. The Levene’s test in all the dimensions are insignificant implying that the equality of variances is verified and the insignificant p-value presented in the t-test concludes that no statistical difference prevails among gender, civil status and students’ perception of CSR thereby rejecting the hypothesis 2.4 & hypothesis 2.5.

## Discussion & conclusions

A primary goal of this study was to see if students' perspectives of CSR were different from one another, and a preliminary examination of the means of the students' replies concerning perceptions suggested that there were really two unique groups of students' perceptions. Individuals may be roughly divided into two groups: those who did better overall and who tend to have more positive views of CSR, and those who did worse overall and who reject these views.

Accordingly, the analysis is expected to support the first hypothesis, "Students' perceptions about CSR can be considered in different dimensions," which affirm the outcomes of the literature review, particularly regarding, that students are mindful of morally as well as socially accountable practises/strategies but are not necessarily making responsible decisions and assert behaviour that contribute to the betterment of community ( Assudani *et al.,* 2011). Factor analysis findings, which delineated three main aspects in students' opinions of CSR, confirm the findings that Students’ perceptions about CSR can be considered in different dimensions.

According to the available data, most students have favourable opinions on sustainability and CSR initiatives ( Bahaee *et al.,* 2014). High social perceptions are associated with CSR because the new age of human values is linked with things like harmony, standard of living, environmental considerations, care for and concern for others' well-being ( González-Rodríguez *et al.,* 2013). Our first derived dimension, pro-CSR attitudes, has a mean score of 5.16, corroborating findings from the studies that CSR is seen as a crucial vital decision, providing growing and positive prequisite to businesses ( Mcguire *et al.,* 1988) which is effective for organizations eminence ( Gallardo-Vázquez and Sanchez-Hernández 2014).

There are certain less favourable impressions of CSR that may drive people into adopting a poor consciousness while processing CSR information. This is since consumer cynicism has a significant repercussion on the attitudes and perceptions of customers ( Connors *et al.,* 2017). The second component addresses attitudes that call into question the significance of CSR and address beliefs that are resistive to CSR. According to the findings of Wymer and Rundle-Thiele, 2017, about only one third of the universities are offering courses that are related to the sustainability. This might be related to the notion that HEI's implementation of a responsible education framework is still in its early stages. Students may not be aware of this problem due to the fact that there is a significant lack of expertise amid educators regarding the value of CSR, their unpreparedness for educating ethics and doubt their ability to influence how students make moral decisions ( Wymer and Rundle-Thiele, 2017).

In the third and final component, company prioritisation is discussed. While it's true that offering products or services with a solid reputation may provide a business an edge in the marketplace, ( Brei and Böhm, 2011), the responses to this component place corporate social responsibility in a secondary plan, in the background, with an average score of 4.68. Many of these students believe that corporate social responsibility (CSR) should take a back seat; a socially conscious business, in their eyes, is one that puts its money where its mouth is in terms of helping people and the planet; as a result, the principal management policy of maximisation of wealth is called into question by the expenses associated with socially conscious conduct ( Wymer and Rundle-Thiele, 2017). Decisions are skewed in favour of CSR because managers are motivated by a pursuit of power, control, and riches rather than by considerations of ethics and social responsibility. Therefore, CSR is given top priority only if it is expected to generate a financial return and advance the company's objectives ( Giacalone and Thompson, 2006).

With regard to the study's secondary goal, which is to determine how students' demographic information affects their views on CSR, differentiation based on age, level of education, and professional experience was supported by the data in this study, which strongly indicates that students' CSR perceptions is influenced by the sociodemographic variables which is in line with the findings of ( Almutawa and Hewaidy, 2020) and is in contradiction to the findings of ( Anand and Singh, 2021; Burcea and Marinescu, 2011; Pätäri *et al.,* 2017; Teixeira *et al.,* 2018; Ugwuozor, 2020). Whereas the t-tests on gender and civil status depicted that there is no statistical relationship between these two variables and the reduced dimensions of students’ impression of CSR which is in line with finding of ( Almutawa and Hewaidy, 2020; Chan *et al.,* 2021).

In a nutshell, we may remark the presence of three main aspects of students' perspectives: i) value CSR, ii) against CSR, and iii) neutral to CSR, and we can claim that positive attitudes to CSR offer the greatest average, suggesting that, generally speaking, students appreciate CSR. Thus, the majority of people agree that CSR is crucial, yet different people have different conceptions about what it entails. What's more, the findings demonstrate that there are no statistically significant variations in students' perspectives on CSR across demographic characteristics including age, gender, educational attainment, and professional experience.

The management implications of this study for higher education institutions are substantial. In their roles as future workers, business owners, and customers, students play an essential role in assessing the effectiveness of CSR actions. Distributing sustainable production and consuming practises will influence transitions toward a more sustainable activity ( Pülzl *et al.,* 2014), and the youths have the potential to be extremely major Influencers on society ( Pätäri *et al.,* 2017). It is of the utmost importance to comprehend and foresee how the students' moral and environmentally conscious actions will develop in their future roles as staff members, managers, and business owners, as well as in the context of their customer roles.

The quantity of participants in the study is one of the constraints of the study as HEIs considered for the data collections were limited to a specific region of the country and the sample size may be considered too little to draw any firm conclusions. Therefore, to have a better perspective of whether the hypotheses that were provided in this work would lead to different results, potential future research may entail a sample that is both more complete and more varied. This would require extending to include new HEIs as well as students of varying ages and regions.

## Data Availability

Zenodo: Corporate Social Responsibility & Students’ Perceptions: Evidence from Indian Higher Education Institutions. https://doi.org/10.5281/zenodo.8328583 ( Sunder, 2023). This project contains the following underlying data:
- Blank Instrument.doc - Coding File.csv - Data File.csv - Raw Data.csv -
Table 1 - Methodology -
Table 2 - Affirmations on CSR under study -
Table 3 - Characterization of the Sample -
Table 4 - Factor Analysis Results -
Table 5 - Validation of the assumptions of MANOVA Data are available under the terms of the Creative Commons Attribution 4.0 International license (CC-BY 4.0).
