# Supplementary material for: Corporate social responsibility & students’ perceptions: evidence from Indian higher education institutions
Source: F1000Res. 2023 Sep 22;12:1187. [Version 1] doi: 10.12688/f1000research.137572.1 (PMC10966334; doi:10.12688/f1000research.137572.1)
Supplement: Peer review history

# Reviewer report: Reviewer response for version 1

**DOI:** 10.5256/f1000research.150737.r225196

**Published:** 2024-03-26

**Authors:** Partakson Romun Chiru

**Affiliations:** 1 Department of Commerce, SRM University Sikkim, Sikkim, India

Title: Corporate Social Responsibility & Students’ Perceptions: Evidence from Indian Higher Education Institutions

 Relevance and Significance:

 The research article addresses a pertinent topic concerning the perceptions of students on Corporate Social Responsibility (CSR) within Indian Higher Education Institutions (HEIs). Given the growing importance of CSR in the corporate landscape and the role of HEIs in shaping future business leaders, understanding students' perceptions holds significant relevance. By investigating how sociodemographic factors influence these perceptions, the study offers valuable insights that can inform CSR education and practice in India.

 Comprehensive Literature Review:

 The article provides a thorough literature review on CSR, HEIs, and students' perceptions, demonstrating a clear understanding of the research context. It covers key theoretical frameworks, empirical studies, and conceptual discussions, thereby providing a comprehensive background for the study. The review effectively sets the stage for the research by highlighting the gaps and emphasizing the need for further investigation.

 Clear Research Question and Objectives:

 The research question and objectives are clearly articulated, focusing on exploring students' perceptions of CSR and examining the influence of sociodemographic factors. This clarity helps guide the study's direction and ensures alignment between research aims and methodologies. The objectives are specific, measurable, and relevant, contributing to the overall rigor of the research design.

 Integration of Concepts:

 The article effectively integrates concepts from CSR literature, educational theory, and sociodemographic research to construct a robust analytical framework. By drawing on established models such as the PRESOR scale and adapting them to the Indian context, the study demonstrates a thoughtful approach to theoretical integration. This integration enhances the validity and applicability of the research findings.

 Identification of Research Gaps:

 The literature review skillfully identifies gaps in existing research, particularly regarding students' perceptions of CSR in Indian HEIs. By highlighting the need for empirical investigation and the lack of studies addressing sociodemographic influences, the article contributes to knowledge development in the field. The research aims to fill these gaps by providing empirical evidence and addressing the identified research questions.

 Broader Implications:

 The study's findings have broader implications for CSR education, organizational practice, and policy development in India. By understanding students' perceptions and the factors influencing them, HEIs can tailor their curriculum and initiatives to promote ethical leadership and social responsibility. Moreover, organizations can leverage these insights to enhance their CSR strategies and engage with future stakeholders effectively.

 Recommendations for Future Research:

 The article offers insightful recommendations for future research, including expanding the sample size, incorporating diverse geographical regions, and examining longitudinal trends. By suggesting avenues for further investigation, the study encourages scholars to build on its findings and advance knowledge in the field of CSR and education. Additionally, exploring the long-term impact of CSR education on students' attitudes and behaviors could provide valuable insights for both academia and practice.

 Overall, the research article demonstrates a strong theoretical foundation, methodological rigor, and relevance to current debates in CSR and education. With its clear research objectives, comprehensive literature review, and insightful findings, the study makes a significant contribution to the scholarly discourse on CSR perceptions in Indian HEIs.

Is the work clearly and accurately presented and does it cite the current literature?

Yes

If applicable, is the statistical analysis and its interpretation appropriate?

Yes

Are all the source data underlying the results available to ensure full reproducibility?

Yes

Is the study design appropriate and is the work technically sound?

Yes

Are the conclusions drawn adequately supported by the results?

Yes

Are sufficient details of methods and analysis provided to allow replication by others?

Yes

Reviewer Expertise:

Corporate Social Responsibility, Finance and Accounting and Marketing

I confirm that I have read this submission and believe that I have an appropriate level of expertise to confirm that it is of an acceptable scientific standard.

# Reviewer report: Reviewer response for version 1

**DOI:** 10.5256/f1000research.150737.r209451

**Published:** 2024-03-03

**Authors:** VJ Byra Reddy, Veena Kallembella

**Affiliations:** 1 Chanakya University, Bangalore, Karnataka, India; 2 Business, Dayananda Sagar Business Academy, Bangalore, Karnataka, India

The study is very well conceptualized and studying the perception of young business students on 'corporate social responsibility' who would don the roles in corporate corridors for the next 30-40 years is very salient. The hypotheses formed and tested appear to be consistent with the objectives of this study. What is very appreciable throughout the study is the 'robust' review cited in all areas of the study and not just the review of literature. Moreover, the literature is current and appropriate. The methods and methodology followed does complete justice to the objectives of the study. 

 However:

1.  The study may need to change its title from the current - Corporate social responsibility & students’ perceptions: evidence from Indian higher education institutions – to - Corporate social responsibility & students’ perceptions: evidence from Indian higher education institutions from **Northeast India**. It is very important to change the title to the effect of the suggestion as the sample collected is from Northeast Indian higher educational institutions. The current title is misleading in the sense that the prospective readers and particularly the research fraternity including research scholars searching this article going by its current title would think that, this study is from the Indian higher educational institutions instead of from where this study actually is from – Northeast India.
2. A question which maybe asked to the researchers is, how using an instrument developed for studying the perception in non-Indian context, answers questions regarding student perception in the Indian context?

Is the work clearly and accurately presented and does it cite the current literature?

Yes

If applicable, is the statistical analysis and its interpretation appropriate?

I cannot comment. A qualified statistician is required.

Are all the source data underlying the results available to ensure full reproducibility?

Yes

Is the study design appropriate and is the work technically sound?

Yes

Are the conclusions drawn adequately supported by the results?

Yes

Are sufficient details of methods and analysis provided to allow replication by others?

Yes

Reviewer Expertise:

Inequalities, Development, Third Sector and Rights and Responsibilities.

We confirm that we have read this submission and believe that we have an appropriate level of expertise to confirm that it is of an acceptable scientific standard.
